# Development of a Soft Robotics Module for Active Control of Sitting Comfort

**DOI:** 10.3390/mi13030477

**Published:** 2022-03-20

**Authors:** Tjark Roozendaal, Martin Verwaal, Alice Buso, Rob B. N. Scharff, Yu Song, Peter Vink

**Affiliations:** 1 Faculty of Industrial Design Engineering, Delft University of Technology, Landbergstraat 15, 2628 CE Delft, The Netherlands; tjark.r@gmail.com (T.R.); m.verwaal@tudelft.nl (M.V.); a.buso@tudelft.nl (A.B.); rob.scharff@iit.it (R.B.N.S.); y.song@tudelft.nl (Y.S.); 2 Bioinspired Soft Robotics Laboratory, Istituto Italiano di Tecnologia, Via Morego 30, 16163 Genoa, Italy

**Keywords:** soft-robotics module, comfort, seat, pneumatics, adaptive

## Abstract

Sitting comfort is an important factor for passengers in selecting cars, airlines, etc. This paper proposes a soft robotic module that can be integrated into the seat cushion to provide better comfort experiences to passengers. Building on rapid manufacturing technologies and a data-driven approach, the module can be controlled to sense the applied force and the displacement of the top surface and actuate according to four designed modes. A total of 2 modules were prototyped and integrated into a seat cushion, and 16 subjects were invited to test the module’s effectiveness. Experiments proved the principle by showing significant differences regarding (dis)comfort. It was concluded that the proposed soft robotics module could provide passengers with better comfort experiences by adjusting the pressure distribution of the seat as well as introducing a variation of postures relevant for prolonged sitting.

## 1. Introduction

Traveling long distances by car, train, or airplane requires sitting in a restricted posture for more extended periods, resulting in discomfort in some body parts. Sammonds et al. [1] showed that discomfort in the human body increases over time. Researchers and designers have tried to make the seat as comfortable as possible, e.g., by following the identified optimal pressure distribution in a car seat [2]. However, the optimal pressure distribution only can delay but not prevent the development of discomfort. Another possible intervention that can significantly decrease discomfort is introducing a walking break of 10 min during prolonged sitting [1]. Though this is not always possible due to practical constraints, it implies that introducing new physical or psychological stimuli might help passengers regain their comfort during prolonged sitting.

Introducing new stimuli to the seat requires functions of both sensing the discomfort level of passengers and actuating accordingly. Sensing the pressure distribution on the seat cushion of the driver or passenger could provide early signals concerning discomfort, as pressure distribution and its changes are closely associated with postures and discomfort [3]. ‘Smart cushions’ that check the posture are already available. One example is the Sensimat [4], a cushion designed for wheelchairs to monitor the sitting activity of the user and provide feedback in order to prevent pressure ulcers. For actuation, Van Veen [5] showed that small continuous movements of seated passengers positively affected objective and subjective indicators of well-being. Researchers also worked on integrating the sensing and the actuation system, e.g., a monitoring system verifying this ideal pressure distribution and, when necessary, altering the seat to satisfy this distribution [6]. However, the mechanism is heavy, and the control system is complicated, which makes it difficult to implement it in vehicles/air planes.

As an alternative to such traditional robotic solutions, the emerging field of soft robotics focuses on developing sensors and actuators built from soft materials [7]. Such actuators have virtually infinite degrees of freedom that allow them to adapt their shape to the objects under interaction. This implies that with the use of pneumatic soft robotics, the actuators can be fabricated in such a manner that they can comply with the forms of the users for a large contact area and more uniform pressure distribution. In addition, it could be more lightweight. This quality has been proven to influence comfort ratings in long-term static sitting [8]. In a car, this would mean that both the pressure distribution between the seat and buttocks can be regulated, e.g., enlarging the contact area between the chair and the user’s contour.

Besides actuation, sensors can also be integrated into the same soft robotics module. Several soft pneumatic robotic combinations of pressure sensors and actuators already exist [9]. Robertson et al. [10] made a soft robotics surface with integrated force-sensitive resistors (FSRs). In addition to contact sensors, scholars introduced contactless sensors, e.g., using optoelectronics [11,12] or electromagnetic fields [13]. An example is the soft actuator module developed by Buso et al. [14] that can use optical sensors to measure the displacement of the actuation and regulate the air intake accordingly to provide a precise displacement. However, the consistency of the manufacturing process of the individual modules needs to be developed, and the control strategy and the feasibility of using it in practical applications remain unknown.

In this paper, we present a soft robotics module that can be integrated into a seat for a better comfort experience for the passenger. The major contributions are: (1) we developed the soft robotics module with embedded sensors, actuators, and control systems; (2) we integrated the module in a seat for providing the ideal pressure distribution both in a normal sitting scenario and in a discomfort scenario; and (3) we tested the seat with 16 subjects to verify the effectiveness of the system.

In the remainder of this paper, Section 2 describes the hardware setup of the soft robotics module. Section 3 further investigates the relations between the displacement, the velocity of the displacement, the force applied on the top of the module, and the sensor readings, using contactless sensors and the data-driven method. Two modules are integrated into a seat (Section 4), and the functionality and practicality of the seat are evaluated by a practical user test (Section 5). The advantages and limitations of the system are discussed in Section 6, and finally, a short conclusion is drawn.

## 2. Components of the Soft Robotics Module

**Figure 1:**
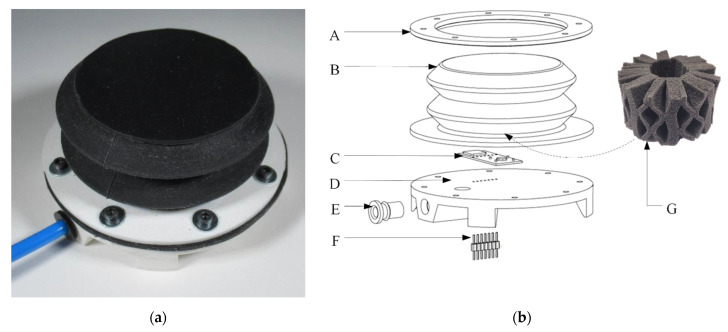
The soft robotics module. (**a**) The module; (**b**) exploded view of the module with A—ring, B—bellow, C—sensors breakout, D—base plate, E—pneumatic fitting, F—male header, and G—Octaspring.

The soft robotic module is developed based on the work of Buso et al. [14]. Figure 1 presents the module (Figure 1a) and an exploded view of its design (Figure 1b). With pneumatic actuation and embedded sensors, the module is able to change its shape and stiffness. The mechanical part of the module consists of a 3D-printed base plate (D in Figure 1b) for housing the pneumatic fittings (E), the male headers (F), and the sensor breakout (C). Moreover, a bellow (B) is fixed on the base plate using the ring (A) and eight M3 screws and nuts. The male header (F) is glued at the bottom of the housing to connect other electronic components outside the module while securing an airtight connection. The bellow (B), a foam spring (Octaspring, Vanema, Slovenia) (G), and the sensor breakout (2) are the core components of the module and will be detailed in the following two sub-sections.

### 2.1. The Bellow

**Figure 2:**
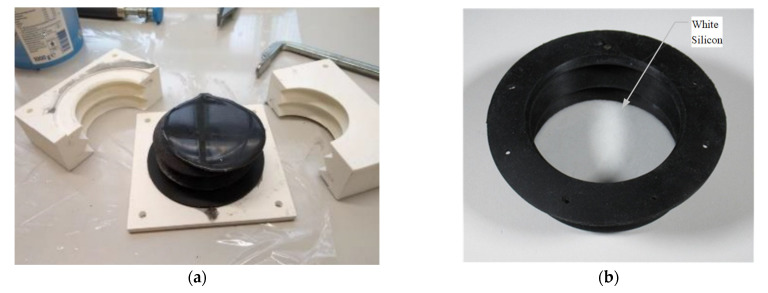

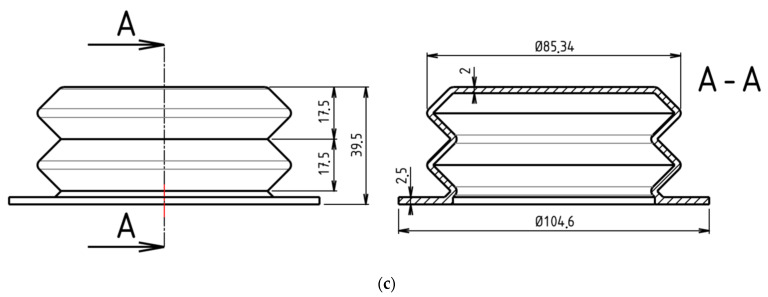
Manufacturing process of the module (left: casting, right: part of the inside surface). (**a**) Mold, (**b**) white inside, and (**c**) dimensions of the bellow (in mm).

The bellow is fabricated by molding Dragon Skin™ 30 silicone (Smooth-On, Macungie, PA, USA) in 3D-printed polylactic acid (PLA) molds, as the left picture in Figure 2a. Once set in the desired shape, the silicone will always return to its original shape after deformation. The geometry of the bellow is designed to transform the changes of the air pressure inside the bellow to linear movements of the top surface of the bellow. Black pigment is used to color the silicone to avoid external light being captured by the optical sensors. To amplify the reflective optical signals inside the bellow, a layer of white silicone is molded in the upside of the inner surface of the bellow (Figure 2b). The dimensions of the bellow are presented in Figure 2c. A full stroke and the travel of the top surface is 30 mm. As the 100% modulus of the silicone is as soft as 0.59 MPa, a piece of Octaspring foam is placed inside the module (G in Figure 1b) to strengthen the stiffness of the module and make it partly functional in case there is a leakage.

### 2.2. The Sensor Breakout

**Figure 3:**
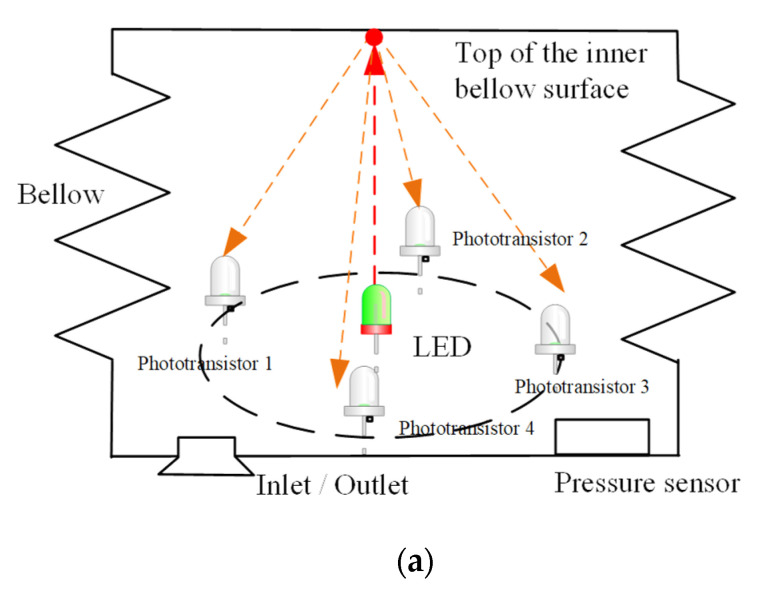

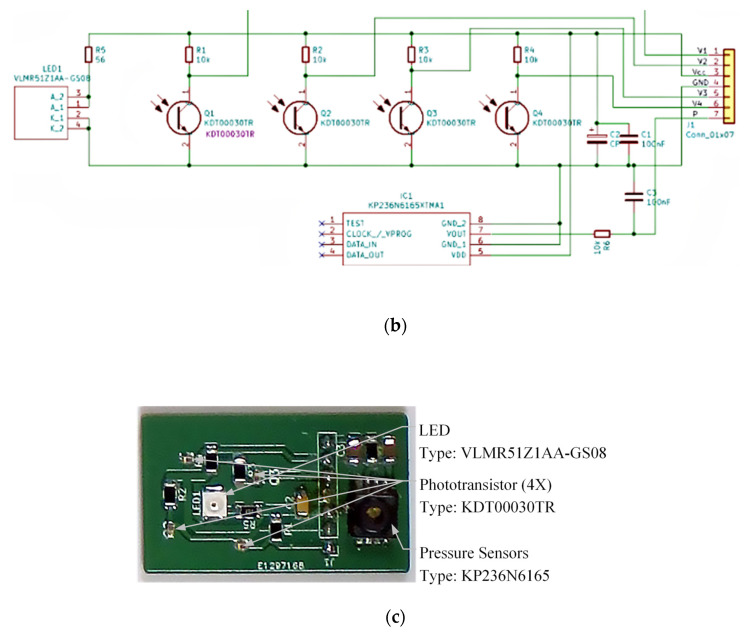
Details of the sensor breakout. (**a**) Principle of the sensor; (**b**) principle of the sensor; (**c**) the sensor breakout.

Figure 3a illustrates the principles of the embedded sensor of the module. At the bottom of the bellow, an inlet/outlet is connected to the pneumatic pump for inflation/deflation of the module. The pressure inside the bellow is measured by a pressure sensor. At the center of the bottom of the bellow, an LED is installed. The light it emits is directed toward the top of the inner surface of the bellow as the red arrow in the figure. As the top inner surface is made of white silicone and the surface finishing is matte, the reflection of a red LED light is a diffused reflection, i.e., the ray incident on the surface is scattered at many angles as the orange arrows in the figure. Four phototransistors, which are positioned around a virtual circle (7 mm radius) of the LED, capture the reflecting lights. In general, the shorter the distance between the LED and the top inner surface is, the higher the intensity of the reflecting light is.

Following the principles, the schematic of the sensor breakout is designed as Figure 3b. The breakout is presented in Figure 3c. In the breakout, a red LED (type: VLMR51Z1AA-GS08, Vishay Semiconductors, Malvern, PA, USA) is selected. In the design of the assembly of the breakout and the bottom plate of the module, the position of the LED is aligned to the center of the bottom of the bellow. Around the LED, four phototransistors (type: KDT00030TR, Onsemi, Phoenix, AZ, USA) are installed, and the absolute air pressure sensor (type: KP236N6165, Infineon, Munich, Germany) is located at the right of the breakout.

## 3. Data-Driven Control of the Module

### 3.1. Actuation of the Bellow

**Figure 4:**
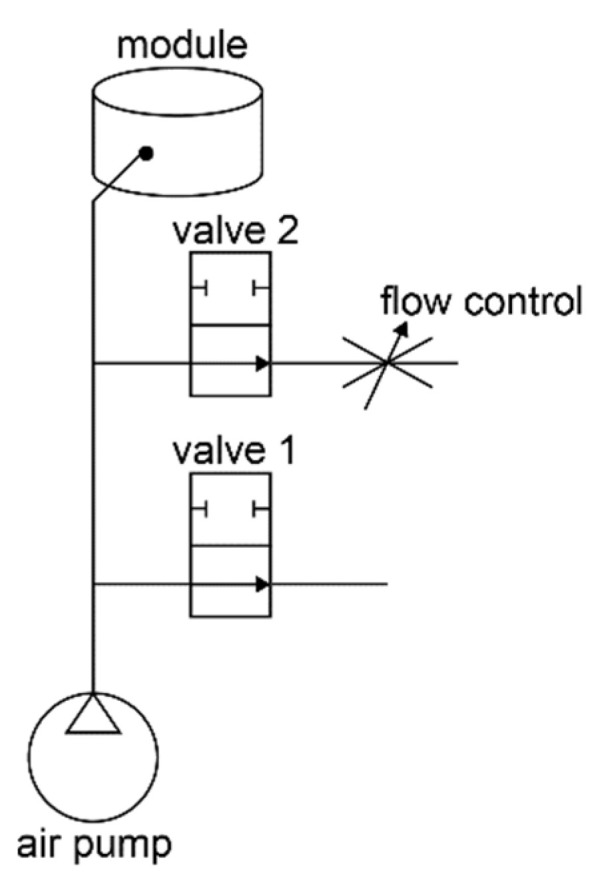
Pneumatics of the module.

One pump and two valves, connected to an electrical power supply and controlled by an Arduino compatible embedded system, are used for actuating each module and maintaining a certain bellow shape, as shown in Figure 4. In the system, valve 1 is open to the atmosphere. Valve 2 had flow control, meaning that if it was open, the air could only escape slowly. Together with the pneumatic pump with varying flow (using pulse width modulation), the system can perform the following actions:

- Action Inflation: In this case, both valves are closed, and the pneumatic pump is switched on. The pressure is measured by the on-board pressure sensor of the sensor breakout; the displacement of the actuation can be measured by the LED and the four phototransistors in the sensor breakout;
- Action Compression: Valve 1 is open and Valve 2 is closed, the displacement and the velocity of the deflation are measured by the LED and the four phototransistors in the sensor breakout; the force can be predicted with use of the pressure sensor;
- Action Balance: Maintaining a certain pressure is crucial for achieving the ideal pressure distribution of the seat cushion. In the use of the module, the force that applied on the top of the surface might differ while be used by different users, and for the same user, tiny movements, i.e., fidgeting, might also lead to changes in the force, which subsequently leads to changes of the shape of the bellow. To maintain the ideal pressure distribution, the dynamic balance is achieved by opening only valve 2 and switching on/off the pneumatic pump based on the measured pressure. Meanwhile, as the material of the bellow is soft silicon, the shape of the bellow changes with the changes of the force applied on the top. In this case, the displacement of the top surface is monitored by the four phototransistors measuring the reflected light of the LED.

**Figure 5:**
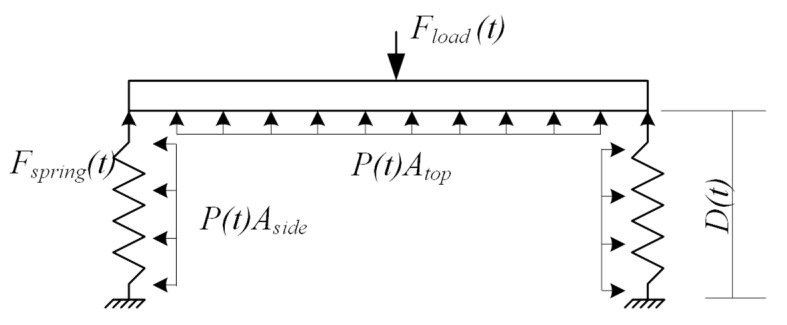
A simplified free-body diagram of the module.

In a mechanical setup of a piston and a cylinder, the force applied to the cylinder can be acquired by $F_{load}\left(t\right)=P\left(t\right)A_{top}$, where $F_{load}$ is the load, P(t) is the pressure applied on the piston, and A is the area of the piston. Assuming that the temperature and the area of the cross-section of the cylinder are constant, displacement D(t) of the piston can be calculated as $D\left(t\right)=P_{0}D_{0}/P\left(t\right)$ using Boyle’s law, where $P_{0}$ and $D_{0}$ are the initial pressure and displacement, respectively. However, the relation between D(t) and P(t) of the soft robotics module are different, as in the free-body diagram presented in Figure 5. In the figure, it can be found that: (1) the side of the bellow is made of silicon and force $F_{spring}\left(t\right)$ is introduced when the bellow is inflated/deflated from the neutral state, i.e., $F_{spring}\left(t\right)=k\left(D\left(t\right)-D_{o}\right)$. Here the value of the spring constant k is related to the geometry, the material, and the manufacturing process of the bellow as well as the Octaspring embedded inside the bellow. In many cases, k is not a constant value regarding the displacement; (2) the changes of P(t) will lead to changes in the geometry of the side surface of the bellow; therefore, the diameter of the bellow is not constant as well.

### 3.2. Data-Driven Approach

**Figure 6:**
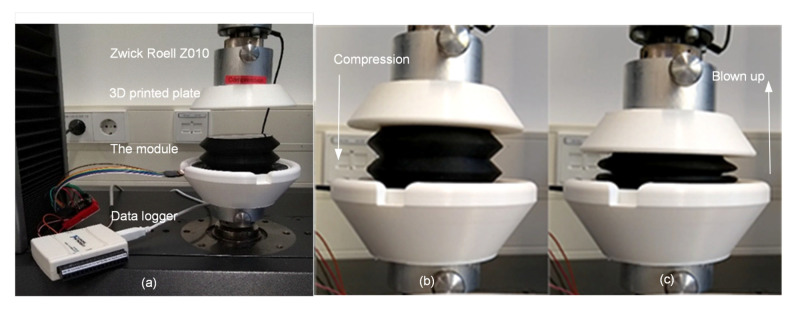
Data collection for the module: (**a**): the setup; (**b**): compression; and (**c**): blown up.

As the geometry of the bellow and the embedded Octaspring is complicated and deviations in the manufacturing process are inevitable, we introduce a data-driven approach for estimating $F_{load}$ and D(t) based on the signals received by the pressure sensor and four phototransistors. Data were collected using a Zwick Roell Z010 machine (ZwickRoell Austria, Ulm, Germany), as in Figure 6a. The loadcell of the Zwick has a maximum capacity of 500 N. Two 3D-printed plates were attached to both ends of the load cell to ensure full contact with the module. A data logger was used to collect analogue signals from the four phototransistors and the air pressure sensor. These five separate sets (four phototransistors and one air pressure sensor) were collected in a table with the corresponding values of the Zwick sensors (force and displacement).

**Figure 7:**
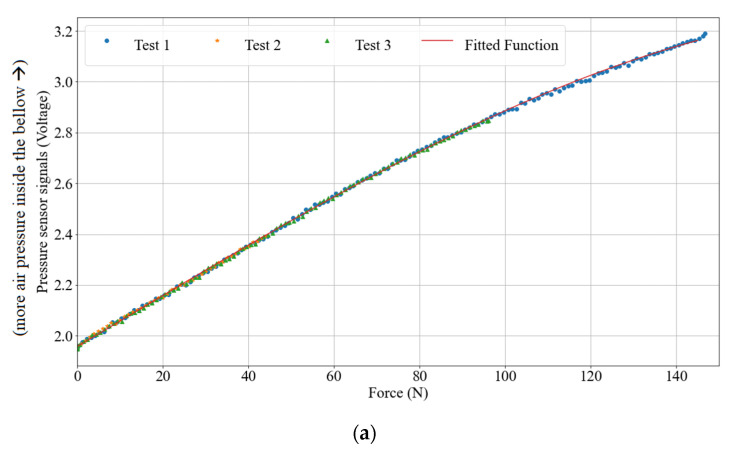

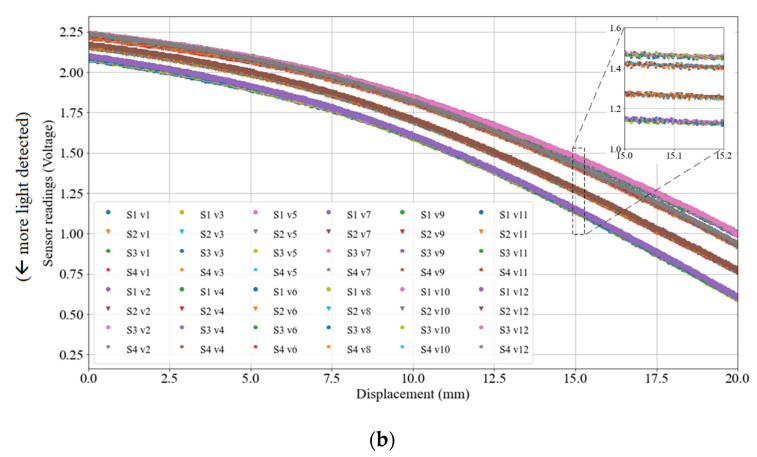
Data collected from the sensors. (**a**) Relations between pressure sensor signals (in V) and the force (in N). (**b**) Relations among displacement, sensor signals (S1 to S4, in V), and the speed (v1 to v12, in mm/s).

In the first experiment, we investigated the relation between the pressure and the force via Action Inflation. We gradually inflated the bellow three times with 50, 100, and 150 N as maximal loads, respectively. The range of the inflation was controlled in such a way that the Octaspring and the inner top surface were always in contact as the right-most picture in Figure 6. Figure 7a presents the relations between the pressure and the force, and high coherence can be found regarding the output of the three experiments. Based on Figure 7a, we modelled the relations between P(t) and $F_{load}$ by polynomial regression as:

(1) $$F_{load}=f_{lp}\left(P\left(t\right)\right)\;\mathrm{and}\;f_{lp}=\left[\begin{array}{ccc} -9.48^{-8} & 1.66^{-6} & \begin{array}{cc} -1.01^{-2} & \;1.96 \end{array} \end{array}\right]\;\left[\begin{array}{c} x^{3}\\ x^{2}\\ \begin{array}{c} x^{1}\\ x^{0} \end{array} \end{array}\right]$$

In the second experiment, we investigated the relations among the displacement, the velocity of the displacement, and the received phototransistor sensor signals via Action Compression. We compressed the bellow 12 times with different velocities as v = [12.24, 8.27, 4.71, 3.18, 2.41,1.62, 1.22, 0.98, 0.82, 0.70, 0.62, 0.55] mm/s. During the compression, valve 1 (as in Figure 4) was kept open. Even though the valve is open, P(t) inside the bellow during different compression tests differ, mainly due to that the flow resistance is influenced by the outlet flow speed.

Figure 7b presents the relations among displacement and the collected sensor signals (voltages from the readout circuit of the four phototransistors). It can be found that (1) although the phototransistors are distributed around the LED, the signals collected from them are not in the sample amplitude, mainly due to the noise and deviations in the manufacturing and assembly; (2) the relation between the displacement and the amplitude of the signal is not influenced by the speed. To model the relations, we introduced a simple artificial neural network (ANN) with an input layer of four neurons, i.e., the four sensor signals $s_{i}\left(t\right)$, a hidden layer of 10 neurons and an output layer of one neuron, i.e., the displacement D(t). Five-fold cross-validation indicates that by using the proposed network, it is able to predict the displacement with a mean absolute error (MAE) of 0.26 mm. Therefore, relations between displacement and the sensor signals can be described as:

(2) $$D\left(t\right)=f_{ann}\left(s_{i}\left(t\right)\right)\;|\;i=1,2,3,4$$

## 4. Embedding the Module in the Chair

With the found relations among force, displacement, and sensors signals, we are able to precisely control the three actions of the soft robotics module described in Section 3. In an ideal use scenario of seat design, the soft robotic modules will be distributed all over the seat pan to: (1) create the ideal pressure distribution [2, 6], and (2) vary the shape of the seat (by varying the geometry) when the occupant is sitting too long in one position (i.e., when not much variation is recorded by the modules). In this study, we integrated two functioning modules into a cushion to verify this concept.

**Figure 8:**
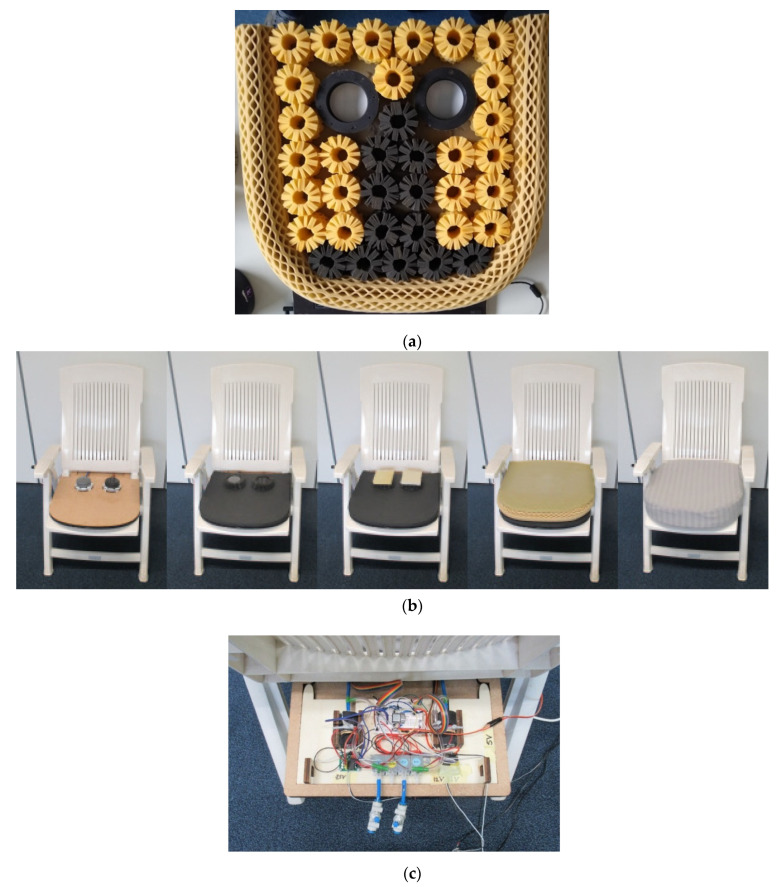
Integration of the soft robotics modules in the chair. (**a**) Octaspring and the space for the soft robotics module in the cushion (as viewed from underneath); (**b**) install the soft robotics module to the chair; (**c**) electronic and pneumatic components positioned under the seat pan.

The design was built on a 520 mm × 520 mm seat cushion from the manufacturer Octaspring (Figure 8a). Pynt et al. [15] explain that in the sitting position, the sitting bones (ischial tuberosities) support most of the body weight. Therefore, two modules were placed in the area where the sitting bones were expected. The cushion with integrated modules is placed on a garden chair to simulate a simplified car seat (Figure 8b). The seat has a backrest angle of 110 degrees and the seat pan 15 degrees, comparable with many automotive seats [2].

Using the sensors signals, it is possible to predict the force and displacement in the inflation, deflation, and balance these three actions. Based on this information, we designed four modes for using the soft robotics modules in the seat as:

(1) Neutral mode, in which all valves are opened so the modules function similarly to the surrounding foam springs;
(2) Blown-up mode, where the module is filled with air and all valves are closed when the subject sits down. This mode creates more pressure on the sitting bones than sitting down in neutral mode because the module cannot be deflated;
(3) Pulsating mode, which starts in neutral mode and inflates and deflates slowly several times (pulsating). The pulsating mode is programmed to smoothly add and subtract 50 N of pressure per module on top of the neutral level (the force that exists when the user is sitting down in the chair without actuation) in a sinus wave fashion over the course of 20 s;
(4) Self-chosen mode, which allows the subject to regulate the pressure added by the modules (0–75 N) by turning a knob. The subject is asked to find the most comfortable position by experimenting with pressure control. The added force at that moment and the neutral force are documented by the researcher.

In the implementation, an embedded system is used to sense and control the actions of a module following the four modes. Each module is equipped with a Seeeduino Xiao (Seeed, Shenzhen, China) that runs two sensing algorithms in a loop, one for predicting the force and one for predicting the displacement. The acquired information is continuously communicated to a central Arduino, which controls the modules following the desired mode. The actuation per module is performed by (de)activating a pneumatic pump and/or two valves, as in Figure 8c.

## 5. Experiment

To explore the optimal parameters of using the proposed soft robotics module in the seat regarding the comfort experience of passengers, we conducted an experiment with 16 subjects. All test subjects are students or employees from the Faculty of Industrial Design Engineering at the Delft University of Technology. A total of 6 of them were female (age: 24–32 years; height: 168–186 cm; body mass: 55–90 kg), and the remaining 10 were male (age: 25–42 years; height: 170–189 cm; body mass: 63–110 kg).

In the experiment, the researcher first welcomes the subject. Then, each subject is asked to sit in the chair four times to experience the four modes specified in Section 4. After each try, the subject is asked to provide a comfort rating (0–10). The subject is then asked to indicate the comfort level regarding each area of the body using a body map [16,17], inspired on the one developed and evaluated by [18]. The same procedure is followed with a discomfort rating except in experiencing the pulsating and self-chosen modes, where the user is asked to pinpoint the moment that they feel most comfortable. The researcher documents the force at that specific moment as well as the neutral force.

Additional questions are asked during testing the modes to evaluate how the (dis)comfort is influenced and can be improved. During the last part of the test, the subject is asked several general questions about this type of actuation in a car chair, such as: “Would you consider this function for car rides of over one hour?”, “What could be improved in this system”, and “Would you like the computer to control this for you?”. The complete test takes around 20 min.

### 5.1. Comfort Ratings

**Figure 9:**
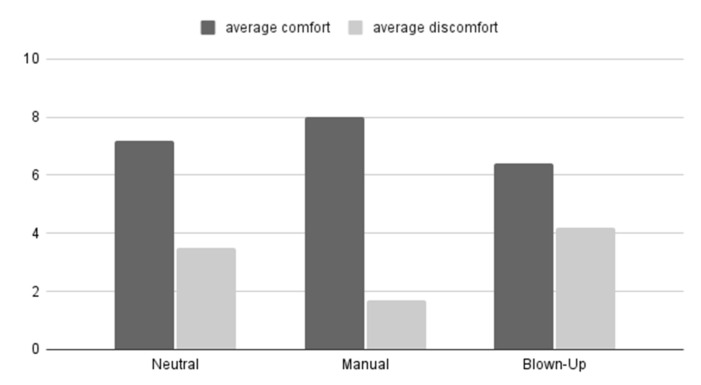
Mean (dis)comfort rating per module on a scale from 0 to 10.

In Figure 9, the average comfort and discomfort ratings during neutral, self-chosen, and blown-up mode are presented. During the pulsating mode, it was found that the subjects had difficulties giving a (dis)comfort rating since the mode was dynamic. For this reason, the (dis)comfort ratings of pulsating mode are not included in the figure.

**Figure 10:**
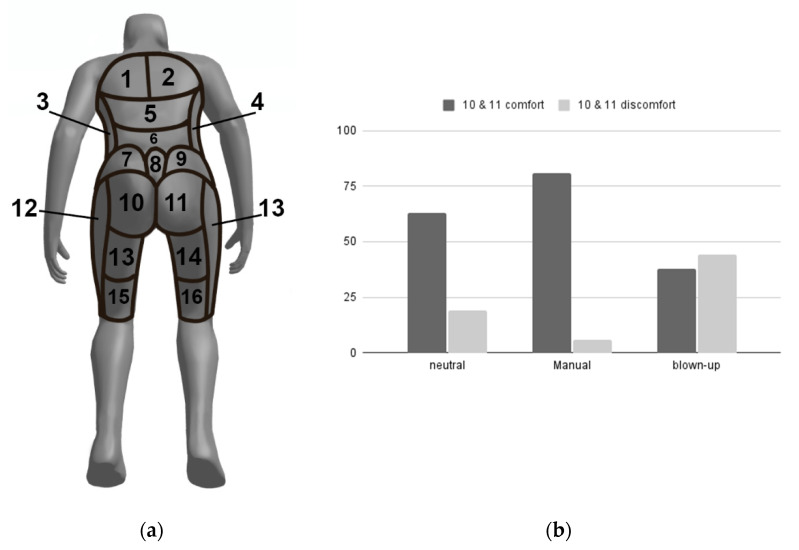
(Dis)comfort areas. (**a**) Body map for indicating the area of (dis)comfort. (**b**) Percentage of most (dis)comfort scores in the buttock area.

When comparing the self-chosen mode (manual) with the neutral mode, the comfort rating is significantly higher (*z* = −3.1798; *p* = 0.00148) for the self-chosen mode and the discomfort rating is significantly lower (*z* = −2.9819; *p* = 0.00288) as Figure 10. Similar results were found when comparing the self-chosen mode with the blown-up mode. The self-chosen mode was perceived more comfortable (*z* = −2.9215; *p* = 0.0035) and showed less discomfort (*z* = −3.4078; *p* = 0.00064).

### 5.2. Area of (Dis)Comfort

Compared to the neutral mode, more subjects mentioned the buttocks area (locations 10 and 11 in Figure 10) in self-chosen mode as comfortable. In the same way, the buttocks area showed less discomfort in self-chosen mode. In blown-up mode, the buttock area was mentioned more often as an area of discomfort than as an area of comfort. This also shows that the participants experienced changes in the buttock area.

### 5.3. Most Comfortable Pressure

During the test, there were two moments when the researcher documented the most comfortable total force on the buttocks of the subject, as measured by the modules. Firstly, during the pulsating mode, the user pinpointed the moment that he/she felt most comfortable. Secondly, during the self-chosen mode, where the user manually adjusted the seat to the most comfortable pressure. In both cases, the neutral level (amount of force when the subject sat down without any actuation), as well as the added force (force that was added on top of the neutral level), were collected.

**Figure 11:**
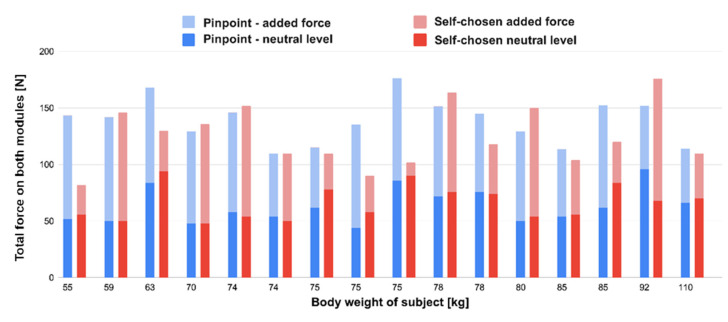
Forces in the chair at the most comfortable moment, per subject, sorted by weight.

Although no obvious trend is visible in this graph (Figure 11), we can notice a relationship between the combination of forces within most subjects. When comparing the different modes per subject, a lower neutral force in pinpoint mode is often accompanied by a higher added force (8 out of 10 cases). Vice versa, a higher neutral force in pinpoint mode implied in half of the occasions a lower added force (2 out of 4 cases). In the remaining two cases, the values were equal.

### 5.4. Actuation Experience

Overall, the system was positively experienced. All subjects preferred such a soft robotic system when driving for more than an hour. All subjects mentioned they would like an automated system, but they also stated that the occupant should be able to overrule the pressure the soft robotic module decides to give. It should be controllable. Adjustability was brought up by 44% of the subjects to be an important factor in creating a combination of manual control and automation. Furthermore, half of the subjects mentioned the personalization of a car seat as a potential use for the system.

### 5.5. Perceived Function of the Modules

During the pulsating mode, 56% of the subjects related the actuation of the modules with a massaging function or feeling. A total of 31% of the subjects said they preferred a lower amplitude in pulsating mode. Furthermore, 38% of subjects indicated during this mode that they needed a simultaneous movement in the (lower) back to compensate the actuation for the seat pan, which highlighted the future work of a “smart backrest”.

## 6. Discussion

Based on the soft robotics module designed by Buso et al. [14], this paper aimed to design and prototype a soft robotics module that can be easily integrated into a seat. The design of the module and the effectiveness of using it in the seat to provide a comfortable experience to passengers are the main focuses.

### 6.1. The Module

In the design and manufacturing process, 3D-printed plates and 3D-printed molds were used to construct the module. The variation among different modules in the manufacturing and assembling process was inevitable. We adopted data-driven design methods to establish the basic control functions. This is in accordance with other studies [11]. Using a data-driven method, the accuracy was acceptable in the context of using the modules in the seat. However, it introduces extra complexity to the control system, and the control parameters differ per module. Moreover, the use of simpler machine learning algorithms such as support vector regression could potentially reduce the number of training samples needed for a reliable training result.

### 6.2. Implications for Seat Design

Zenk [6] proposed that in an ideal pressure distribution of a car seat, 50–65% of the body weight is supported by the buttocks area. Per subject, the most comfortable amount of pressure was recorded in two modules. In pinpoint mode, this was an average of 18.4% of the body weight; in self-chosen mode, this was 16.8%. By integrating more modules into the seat pan, as proposed in, a larger surface can be used to measure forces and adapt the stiffness per module accordingly.

Regarding the pulsating mode, Zenk et al. [19] recommend that seat movement introduced shall be slow, smooth, and small. Making use of the module is promising, and it will be interesting to study how often the form of the seat (arranged by the soft robotic modules) should be changed to create a more comfortable seat or a seat with less discomfort.

### 6.3. Limitations

The two modules built into the seat covered an insufficient area for optimizing the pressure distribution. Moreover, the seat pan used in this work is larger than that in the average car seat. We recommend using a real car seat with a higher number of modules in future studies.

Research indicated that for a passenger sitting in a car, discomfort in the body increases over time. This is why it is interesting to see the effect of the soft robotic modules in a seat on (dis)comfort after sitting for a long time. The user test in this article lasted only 5 min per programmed mode. Sammonds et al. [1] studied seat comfort over two hours and saw that intervention after one hour reduced the discomfort. A study with a longer time period, e.g., >60 min, might reveal more about the effectiveness of the system.

When designing for the user experience in this project, only comfort related to biomechanics was discussed. In future works, it could be interesting to also take thermal comfort into account during longer periods of sitting on a seat [20].

## 7. Conclusions

In this paper, we developed a soft robotic module for a seat pan to offer a better comfort experience to the passengers. The embedded sensors in the soft robotic module are able to provide information to control and displacement as well as the force of the modules. Two modules are built into a seat pan, and participants were able to experience the comfort levels. Experiments results show significant differences regarding (dis)comfort, indicating that the principle works. Further development is needed to make a seat pan with more modules, combined with a central computing system that monitors, records, and regulates the modules. Simplification of the data-driven approach to calculate the force and displacement is recommended, as well as large-scale user tests for a period of more than 60 min.

## Data Availability

Data available on request due to privacy restrictions.
